# Feasibility and acceptability of an electronic decision aid for genetic testing in ovarian and pancreatic cancer patients

**DOI:** 10.1093/rescon/vmag026

**Published:** 2026-04-28

**Authors:** Danielle A Lynch, Sarah Wiser, Devanshi Patel, Kajal Patel, Ava Siegel, Mackenzie Wooters, Linda Rodgers-Fouche, Sara Bouberhan, Colin Weekes, Nana Addo-Tabiri, Kimberly Zayhowski, Elizabeth Pottier, Karen R Sepucha, Yuchiao Chang, Kristen M Shannon, Daniel C Chung

**Affiliations:** Division of Gastroenterology, Massachusetts General Hospital, Boston, MA 02114, United States; Division of Gastroenterology, Massachusetts General Hospital, Boston, MA 02114, United States; Center for Cancer Risk Assessment, Massachusetts General Hospital, Boston, MA 02114, United States; Division of Gastroenterology, Massachusetts General Hospital, Boston, MA 02114, United States; Division of Gastroenterology, Massachusetts General Hospital, Boston, MA 02114, United States; Center for Cancer Risk Assessment, Massachusetts General Hospital, Boston, MA 02114, United States; Center for Cancer Risk Assessment, Massachusetts General Hospital, Boston, MA 02114, United States; Cancer Center, Massachusetts General Hospital, Boston, MA 02114, United States; Cancer Center, Massachusetts General Hospital, Boston, MA 02114, United States; Hematology and Medical Oncology, Boston University Chobanian and Avedisian School of Medicine, Boston, MA 02118, United States; Department of Medical Sciences and Education, Boston University Chobanian and Avedisian School of Medicine, Boston, MA 02118, United States; Hematology and Medical Oncology, Boston University Chobanian and Avedisian School of Medicine, Boston, MA 02118, United States; Division of General Internal Medicine, Health Decision Sciences Center, Massachusetts General Hospital, Boston, MA 02114, United States; Division of General Internal Medicine, Massachusetts General Hospital, Boston, MA 02114, United States; Division of General Internal Medicine, Massachusetts General Hospital, Boston, MA 02114, United States; Center for Cancer Risk Assessment, Massachusetts General Hospital, Boston, MA 02114, United States; Division of Gastroenterology, Massachusetts General Hospital, Boston, MA 02114, United States; Center for Cancer Risk Assessment, Massachusetts General Hospital, Boston, MA 02114, United States

**Keywords:** Genetic testing, Pancreatic cancer, Ovarian cancer, Decision aid

## Abstract

**Background and aims:**

Germline genetic testing is recommended for all patients with ovarian or pancreatic cancer, and alternative service delivery models are needed to meet this demand. We evaluated an electronic decision aid (DA) that can be administered during a visit with an oncology provider. The DA centered around the decision to pursue genetic testing and selection of a specific multi-gene panel.

**Methods:**

An electronic DA had previously been developed by a multi-disciplinary team. Patients were enrolled at two academic medical centers. We surveyed participants with ovarian or pancreatic cancer who completed the DA about their experience and knowledge gained. Oncology providers were also surveyed.

**Results:**

Ninety-two patients were enrolled. The DA took 13.7 min to complete. There was a 20% increase in knowledge scores after using the DA. Ninety-five percent opted to pursue genetic testing; 20% chose the smallest cancer-specific gene panel, 31% chose the mid-size multi-cancer panel, and 49% chose the largest panel that included limited evidence genes. Nearly all participants (93%) indicated the DA helped their decision-making, and 95% would recommend the DA to friends interested in genetic testing. Ninety-one percent of providers felt patients were well-informed about genetic testing options and 93% indicated the DA did not extend appointment times.

**Conclusions:**

Use of a DA for genetic testing is feasible and acceptable. Patients and providers expressed high levels of satisfaction. The wide range of multi-gene panels selected highlights the importance of offering choice. Studies to formally compare the performance of the DA with traditional genetic counseling are warranted.

## Introduction

Although the role and indications for genetic testing for inherited cancer syndromes are rapidly expanding, rates of germline genetic testing for eligible patients continue to fall short. The National Comprehensive Cancer Network (NCCN) began recommending universal germline genetic testing in patients with ovarian cancer in 2014 [1]. This same recommendation was made for all patients with pancreatic cancer by the NCCN in 2018 [2]. However, a 2021 meta-analysis found that the referral rate for genetic counseling for ovarian cancer patients was only 28%–64%, and the genetic testing rate was between 16% and 63% [3]. The low rates of genetic testing are due not only to low referral rates but also long wait times for a genetic counseling appointment, limitations of the genetic counselor workforce, and low patient uptake of genetic testing [2, 4–6].

Pre-test genetic counseling supports the patient’s ability to make an informed decision by educating them about the benefits, limitations, and implications of genetic testing [7]. This foundational element must be maintained when designing any new paradigm for pre-test genetic counseling. Beyond deciding whether to test or not, there is also an important decision about which of many multigene panel options to pursue [8]. Larger panels offer the potential of more information, but at the risk of higher rates of uncertain or unactionable results [9]. These larger panels typically include genes that underlie a broad range of inherited cancer syndromes rather than focusing on genes relevant to a patient’s specific cancer type.

Alternative service delivery models (SDMs) have demonstrated the ability to improve rates of timely genetic testing while maintaining patients’ informed decision-making [6, 10, 11]. Models include tele-genetic counseling, embedding a genetic counselor into a multidisciplinary clinic, partnering genetics and non-genetics providers, mainstreaming genetic testing, and utilizing patient education tools [6, 12, 13]. Decision aids (DAs) are tools that promote shared decision-making and can increase knowledge, clarify patient values, and promote realistic expectations for a range of healthcare decisions [14, 15]. In the cancer genetics setting, DAs have previously been used alongside a genetic counselor [16, 17]. Such a model does not address a barrier many patients face—namely, the difficulty of timely access to an appointment with a genetic counselor. A paradigm that utilizes a DA in the pre-test setting without a visit with a genetic counselor may reduce this barrier and increase access to genetic testing.

A multi-disciplinary team at Mass General Hospital (MGH) previously developed an interactive, electronic DA to support ovarian and pancreatic cancer patients that is uniquely designed to assist in the selection among three multi-gene panel options [18]. We performed a survey study to assess the feasibility and acceptability of this electronic DA by evaluating patient and provider perceptions of the tool and patient knowledge gained.

## Methods

### Participants

Participants were enrolled from two urban, academic cancer centers at Massachusetts General Hospital (MGH) and Boston Medical Center (BMC). BMC is a safety-net hospital with a smaller catchment area. All patients with ovarian cancer or pancreatic cancer at either institution and referred for genetic counseling were screened for eligibility. Patients over the age of 18 were offered the DA at their next oncology visit if they had epithelial ovarian cancer or pancreatic adenocarcinoma and did not have previous germline genetic testing.

### Design

Eligible patients who were willing to complete the DA were recruited by research coordinators (RC) to participate in the survey study. Patients did not have to join the survey study to get access to the DA. Patients were asked to fill out two types of surveys for a total of three surveys to be completed, one knowledge survey given twice (before and after using the DA) and an additional survey after completing the DA to measure the patient’s experience. Providers were asked to complete a provider survey after each visit with an enrolled patient.

### Decision aid development and content

An electronic DA was previously developed by a multi-disciplinary team of physicians, genetic counselors, decision aid scientists, health care communication specialists, and patients to assist patients in cancer genetic testing decisions, including whether they wanted genetic testing and, if so, the type of multi-gene panel to pursue. The specific details of the development of the electronic decision aid have been reported [18]. Briefly, the tablet-based tool consists of 12 pages of text, videos, and interactive questions that guide the patient through six topics about genetics and genetic syndromes, ascertain their values, and support them in deciding about genetic testing. The tool was developed to be used alongside a visit with an oncology provider who would answer any further questions and obtain informed consent for genetic testing. The tool first guided the participant about the decision to pursue genetic testing. For those who chose to proceed, it then guided the participant to choose among three multi-gene panel options tailored to their cancer diagnosis—a small cancer-specific, medium common-cancer, or broad cancer panel. In the context of the decision aid, panel selection was guided by cancer type alone and did not take family cancer history into account. For the options offered to ovarian cancer patients, the small panel included 14 genes specifically linked to ovarian cancer, the medium panel (36 genes) included the 14 ovarian cancer risk genes as well as additional genes related to other common cancers such as breast and colon cancer, and the broad panel (85 genes) contained all of the previous genes as well as rare genes and genes with only preliminary evidence linking them to cancer risk (Supplementary Table S1, see online supplementary material for a color version of this table). The options offered to pancreatic cancer patients were similar. The small panel included 20 genes specifically linked to pancreatic cancer, the medium panel (47 genes) included the 20 pancreatic cancer risk genes as well as additional genes related to other common cancers, and the broad panel (84 genes) contained all of the previous genes as well as rare genes and genes with only preliminary evidence linking them to cancer risk.

### Surveys and outcomes

#### Patient knowledge (pre- and post- DA)

A 10-question, multiple-choice knowledge survey assessed the participant’s understanding of critical principles for making an informed decision about genetic testing (Appendices 1 and 2).

#### Decision aid acceptability (post- DA)

Patients completed a 10-question survey about the length, density, clarity of content, experiences of technical difficulties, ease of use, and their likes, dislikes, and ideas for improvement for the DA (Appendix 3).

#### Provider survey

A 12-question survey given to providers measured their perception of the patient’s decision-making and the impacts of the DA on clinic flow, specifically, visit length. It also gathered what they liked about the DA, any suggestions for improvement, and any questions their patients may have had that the provider felt should have been covered in the DA (Appendix 4).

#### DA usage

Data on how long a patient used the DA (minutes) and how many pages were viewed by the patient were collected through Google Analytics as well as manually by a RC.

#### Testing decision

Data on whether a patient chose a test and which panel they chose were recorded on the DA as the patient clicked through the interactive questions and collected when their decision was printed on a summary sheet for their provider.

Open-ended patient and provider experience survey responses were manually coded into categories for analysis.

Disclosure of genetic test results was not a formal component of this study and was the responsibility of the ordering clinician as part of routine clinical care. Clinical protocols are in place to refer all patients whose test results included a pathogenic variant, likely pathogenic variant, or variant of uncertain significance to a genetic counselor for formal discussion.

### Procedure

A RC approached eligible patients to offer the DA at a future clinic appointment. At that appointment, the RC administered a baseline knowledge survey to interested participants before using the DA. The RC then briefly introduced the tool and left the participant to use the DA. Once the DA was completed, the RC provided the patient with printed take-home materials and informed their provider of the patient’s test choice via a summary sheet. The provider confirmed the participant’s decision and obtained informed consent for genetic testing, if appropriate. The second set of surveys was administered directly after using the DA or later in the day. The length of time participants spent using the DA and which pages were viewed were also recorded. The RC gave the oncology provider a survey after the appointment. For non-English speaking patients, interpreters were used at each step of this process, with the questionnaires and decision aid being translated by a certified translator.

### Statistical analysis

We used descriptive statistics to summarize the demographic and clinical characteristics of study participants. We measured patient acceptability as the percentage who found the DA to be helpful in making a decision about genetic testing and would refer the DA to friends. We evaluated patient feasibility by time spent using the DA and the percentage of patients who reported technical difficulties using the DA. We calculated provider acceptability by the percentage who were very or extremely satisfied with their DA visit.

We compared continuous outcomes (the time spent on the DA, number of pages viewed) between ovarian and pancreatic cancer groups using linear regression models and compared categorical outcomes (declining genetic testing, multi-gene panel choices, ratings of the information presented in the DA) using logistic regression models, controlling for cancer stage. We compared these characteristics between pancreatic cancer patients and ovarian cancer patients using chi-square tests for categorical variables and *t* tests for continuous variables.

To examine preliminary efficacy, we used paired *t*-tests to assess whether there were significant changes in knowledge score before and after DA and used McNemar’s tests to compare the proportion with correct answers before and after DA. We used chi-square tests to compare the responses between ovarian and pancreatic cancer providers. Analyses were conducted using SAS version 9.4 with a two-tailed significance level of 0.05.

The Mass General Brigham Institutional Review Board (single IRB mechanism) approved the study (protocol #2020P003422). The study was registered at Clinicaltrials.gov (NCT04704193).

## Results

### Screening and enrollment

**Figure 1:**
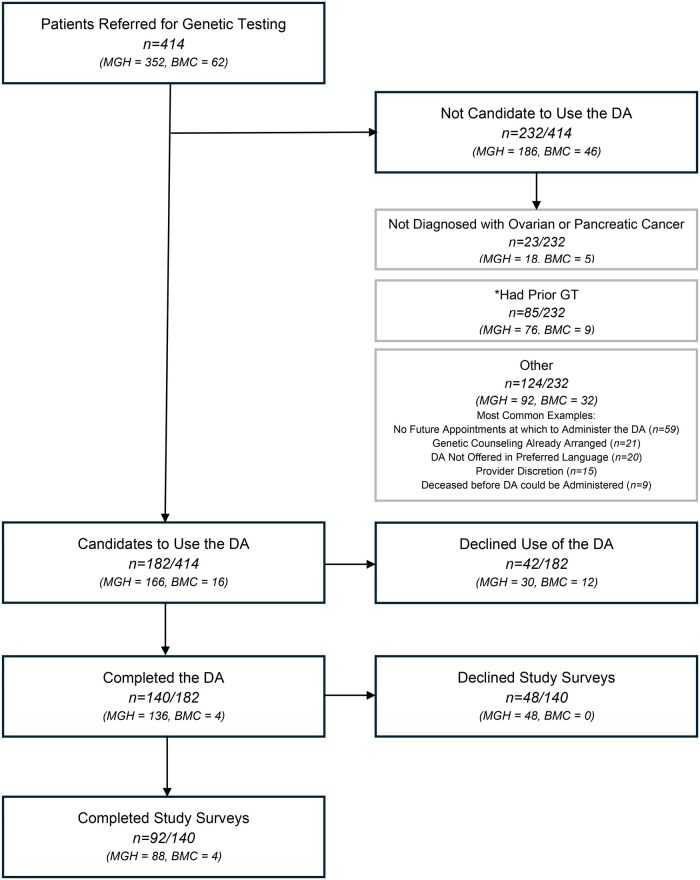
Flowchart of screening and enrollment. A total of 182 ovarian and pancreatic cancer patients were found to be candidates to use the DA during the study period. Of these, 92 completed the DA and study surveys. A total of 88 participants were seen at MGH and 4 participants were seen at BMC. ^a^Five patients were found to not be candidates due to both prior genetic testing (GT) and not having a diagnosis of ovarian or pancreatic cancer. To avoid being counted twice, these participants are included in the “not diagnosed with ovarian or pancreatic cancer” category but not the “prior GT” category. *n* = number of participants in each category. The numbers are broken down in parentheses into the two sites (MGH and BMC). Denominators are shown to indicate the proportion of participants at each step of the flowchart.

Between November 2020 and September 2023, 414 patients (352 at MGH; 62 at BMC) with suspected ovarian or pancreatic cancer were referred for genetic counseling at their respective institutions. After screening patients for DA eligibility, 232 patients (186 at MGH, 46 at BMC) were not candidates to use the DA. Common reasons included incorrect diagnosis at time of referral, prior completion of genetic testing, and lack of a future appointment to administer the DA (Fig. 1). Of the remaining 182 patients found to be candidates to use the DA (166 at MGH; 16 at BMC), 140 patients (136 at MGH; 4 at BMC) opted to complete the DA tool (140/182, 76.9%). There was a significant difference in the uptake of the DA among those offered the DA at the two institutions (136/166, 82% at MGH and 4/16, 25% at BMC, *P* < .001). About 92 of these 140 agreed to participate in the study surveys (88 at MGH; 4 at BMC) and comprised the study cohort.

### Demographics

The demographics for the 92 enrolled participants who completed the study surveys are shown in Table 1. The average age at enrollment was 64.7 (SD 12.5), 86% were white, and 90% were non-Hispanic. While the majority of the participants were English speakers, one participant was a non-English speaker with Spanish being their preferred language. Forty-seven participants (51%) had ovarian cancer, and 45 participants (49%) had pancreatic cancer. Twenty-seven (60%) of the patients diagnosed with pancreatic cancer were male. Aside from sex assigned at birth, the only significant difference between ovarian and pancreatic cancer patients was cancer stage at diagnosis, with the majority of ovarian cancer patients diagnosed at stage 3 (51%), while most pancreatic cancer patients were diagnosed at stage 4 (62%, *P* < .001). The demographics of participants from BMC are shown in Supplementary Table S2 (see online supplementary material for a color version of this table).

**Table 1:** Demographics of participants.

	Total participants	Ovarian cancer patients	Pancreatic cancer patients	*P*-value^a^
	*n* = 92	*n* = 47/92	*n* = 45/92	
**Age (years)**				.311
<50	9 (9.8%)	7 (14.9%)	2 (4.4%)	
50–70	55 (59.8%)	28 (59.6%)	27 (60.0%)	
>70	28 (30.4%)	12 (25.5%)	16 (35.6%)	
**Gender**				<.001
Male	27 (29.3%)	47 (100%)	27 (60.0%)	
Female	65 (70.7%)	0 (0%)	18 (4.00%)	
**Race**				.573
White	79 (85.9%)	43 (91.5%)	36 (80.0%)	
Black or African American	6 (6.5%)	2 (4.3%)	4 (8.9%)	
Asian	3 (3.3%)	1 (2.1%)	2 (4.4%)	
Declined/other	4 (4.3%)	1 (2.1%)	3 (6.7%)	
**Ethnicity**				.946
Non-Hispanic or Latino	83 (90.2%)	42 (89.4%)	41 (91.1%)	
Hispanic or Latino	2 (2.2%)	1 (2.1%)	1 (2.2%)	
Other	7 (7.6%)	4 (8.5%)	3 (6.7%)	
**Cancer stage^b^**				<.001
I	12 (13.0%)	7 (14.9%)	5 (11.1%)	
II	10 (10.9%)	5 (10.6%)	5 (11.1%)	
III	31 (33.7%)	24 (51.1%)	7 (15.6%)	
IV	39 (42.4%)	11 (23.4%)	28 (62.2%)	
**Site location**				
MGH	88 (95.7%)	46 (97.9%)	42 (93.3%)	
BMC	4 (4.3%)	1 (2.1%)	3 (6.7%)	

a Chi-square tests between ovarian and pancreatic cancer patients.

b Staging per AJCC, 8th edition, 2018.

### Participant experience

#### Use of the decision aid

On average, participants completed the DA within 29.9 (±34.7) days of referral with a median (IQR) of 22.5 (14–35) days. Participants completed the DA in the clinic while waiting to see their oncology provider. The DA took between 6.0 and 29.7 min for participants to complete, with an average time of 13.7 min. There were no statistically significant differences in time spent on the DA between ovarian and pancreatic cancer patients (Table 2). Out of the 12 pages featured on the DA, patients viewed between 6 and 20 pages. If a participant viewed more than 12 pages, that participant viewed a page or pages more than once. Ovarian cancer participants viewed an average of 13.5 pages (SD 2.7), while pancreatic cancer patients viewed an average of 11.8 pages (SD 1.9) (*P* = .027) (Table 2). Only 5 (5.4%) of the 92 participants experienced technical difficulties while using the DA (i.e., WIFI connectivity, audio issues).

**Table 2:** Time spent, pages viewed, and technical difficulties reported by participants.

		Total participants	Ovarian cancer patients	Pancreatic cancer patients	*P*-value^a^
**Time spent on DA, minutes**		*n *= 85	*n *= 45	*n *= 40	
	Mean (± SD)	13.7 (± 5.6)	12.9 (± 5.9)	14.6 (± 5.1)	.187
	Median	13.0	11.0	14.1	
	Q1	9.0	8.0	11.0	
	Q3	17.0	18.0	16.2	
	Minimum	6.0	6.0	6.7	
	Maximum	29.7	28.0	29.7	
**Pages viewed (12 pages featured on the DA)** Pages can be reviewed more than once		*n *= 77	*n *= 45	*n *= 32	
	Mean (± SD)	12.8 (± 2.5)	13.5 (± 2.7)	11.8 (± 1.9)	.027
	Median	12.0	12.0	12.0	
	Q1	12	12	12	
	Q3	14	14	12	
	Minimum	6.0	7.0	6.0	
	Maximum	20.0	20.0	18.0	
**Reported technical difficulties**		*n *= 92	*n *= 47	*n *= 45	
	Count (%)	5 (5.4%)	3 (6.4%)	2 (4.4%)	.392

a Adjusted for cancer stage.

The DA has interactive questions designed to help clarify the patient’s values and guide their decision-making process. Of the 92 participants, 80 (87%) completed all 10 questions, 3 (3%) completed a subset of the questions, and 9 (10%) skipped all the questions. Each question could be answered on a scale of 1 through 7; lower numbers suggest a stronger preference for no genetic testing, and higher numbers indicate a stronger preference for genetic testing. The average rating was 5.9 (SD 0.9), indicating that most participants’ values aligned with pursuing genetic testing (Supplementary Table S3, see online supplementary material for a color version of this table). There was no difference in the overall score between the ovarian and pancreatic cancer patients (mean 5.8 vs. 5.9, *P* = .689). However, the pancreatic cancer participants ranked their interest in “understanding my risk for other cancers” lower than the ovarian cancer participants (mean 6.3 vs. 6.8, *P* = .011). The small number of participants from BMC precluded a comparison between sites, but the institution-specific scores are presented in Supplementary Table S4 (see online supplementary material for a color version of this table).

#### Testing uptake and panel choice

After using the DA, 94.6% (*n* = 87/92) of participants decided to pursue genetic testing and selected one of the three multi-gene panels. Most of these participants (*n* = 64/87, 73.6%) had their blood drawn for genetic testing on the same day they completed the DA; the remaining participants had a sample collected at a subsequent visit.

**Figure 2:**
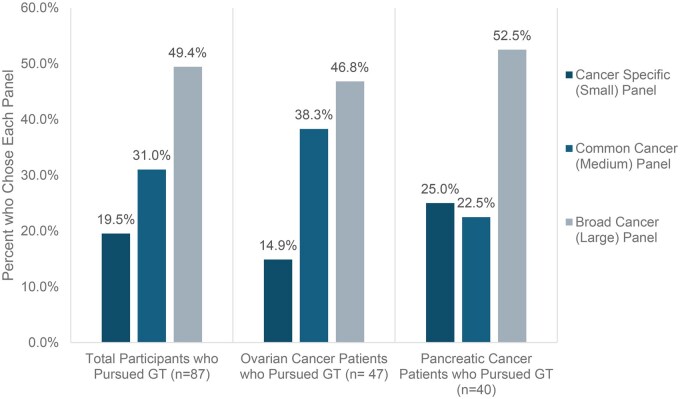
Breakdown of gene panel choice among all participants. After using the decision aid, 87 of the 92 participants opted to pursue GT. Of these 87 participants, 17 participants (19.5%) selected the cancer-specific (small) panel, 27 participants (31.0%) chose the common cancers panel, and 43 participants (49.4%) chose the broad cancer panel, which includes preliminary evidence genes (PEGs). There is no statistically significant difference in choice between ovarian cancer and pancreatic cancer patients. Of the four BMC participants, one (25%) chose the cancer-specific (small) panel, and the remaining three (75%) chose the broad cancer panel. The specific genes in each panel are described in Supplementary Table S1 (see online supplementary material for a color version of this table).

Those who pursued genetic testing exhibited a range of preferences between the three multigene panel choices (Fig. 2). Seventeen (19.5%) participants chose the small cancer-specific panel, 27 (31.0%) participants chose the medium common-cancer panel, and 43 (49.4%) participants chose the broad panel. There were no statistically significant differences in multigene panel choice distribution between the ovarian and pancreatic cancer groups. Out of the remaining five participants who declined to pursue genetic testing after completion of the DA, three requested to see a genetic counselor, while the remaining two declined genetic testing altogether. Of the three participants who requested to see a genetic counselor, two completed testing after their appointment, while one declined scheduling when contacted by the genetics department. Notably, all five of these participants had pancreatic cancer (11.1% vs. 0%).

#### Knowledge

Fifty-two participants (49 at MGH, 3 at BMC) completed both the baseline and follow-up knowledge surveys to assess how much they learned from the DA. Overall, there was a significant increase from an average baseline score of 6.0/10 (SD 1.5) to 7.2/10 (SD 1.5) post-DA (mean increase = 1.2 [SD 1.3], *P* < .001). Supplementary Table S5 (see online supplementary material for a color version of this table) shows the percentage of participants who answered each question correctly at baseline and follow-up time points. Question 4, which measured understanding of an uncertain result, was the only question that showed significant improvement (62% vs. 83%, *P* < .001). There were no decreases in knowledge scores after use of the DA. There were two questions (#6 and #7) in which the percentage of correct responses after the use of the DA was below 50% (Supplementary Table S5, see online supplementary material for a color version of this table).

#### Reported experience

After completing the DA, 86 participants completed at least some of the patient experience survey. Most participants indicated that the length of the DA (*n* = 82/86; 95.3%) and the amount of information presented (*n* = 77/84; 91.7%) were “just right” (Table 3). Out of 83 participants, 55 (66.3%) participants felt that the information given by the DA was balanced, while 28 (33.7%) participants felt the DA “encouraged genetic testing.” Most participants said that the DA helped them make a decision about genetic testing (*n* = 75/81; 92.6%) and would recommend it to friends interested in genetic testing (*n* = 77/81; 95%). Additionally, there were six main topics presented in the DA, as shown in Supplementary Table S6 (see online supplementary material for a color version of this table). When asked to rate the presentation of these main topics, most participants rated the information presented to them as either “good” or “excellent.” Institution-specific patient responses are shown in Supplementary Table S7 (see online supplementary material for a color version of this table).

**Table 3:** Participant responses to the patient survey about their experience using the decision aid by cancer type.

Question	Rating	Total participants	Ovarian cancer patients	Pancreatic cancer patients	*P*-value^a^
		*n* = 86	*n* = 47	*n* = 39	
The length of the decision aid tool was:	Too short	2 (2.3%)	1 (2.1%)	1 (2.6%)	.663
	Just right	82 (95.3%)	45 (95.7%)	37 (94.9%)	
	Too long	2 (2.3%)	1 (2.1%)	1 (2.6%)	
		*n* = 84	*n* = 45	*n* = 39	
The amount of information was:	Too little information	4 (4.8%)	2 (4.4%)	2 (5.1%)	.862
	Just right	77 (91.7%)	42 (93.3%)	35 (89.7%)	
	Too much information	3 (3.6%)	1 (2.2%)	2 (5.1%)	
		*n* = 83	*n* = 47	*n* = 36	
I thought the information presented:	Discouraged genetic testing	–	–	–	.623
	Was balanced	55 (66.3%)	32 (68.1%)	23 (63.9%)	
	Encouraged genetic testing	28 (33.7%)	15 (31.9%)	13 (36.1%)	
		*n* = 81	*n* = 46	*n* = 35	
Did you find the decision aid to be helpful in making your decision about genetic testing?	Yes	75 (92.6%)	43 (93.5%)	32 (91.4%)	.661
	No	6 (7.4%)	3 (6.5%)	3 (8.6%)	
		*n* = 81	*n* = 46	*n* = 35	
Was the final summary of your preferences accurate and clear to you?	Yes	77 (95.1%)	46 (100%)	31 (88.6%)	.933
	No	4 (4.9%)	–	4 (11.4%)	
		*n* = 81	*n* = 46	*n* = 35	
Would you recommend this decision aid to friends who are interested in genetic testing?	Yes	77 (95.1%)	43 (93.5%)	34 (97.1%)	.508
	No	4 (4.9%)	3 (6.5%)	1 (2.9%)	

a Adjusted for cancer stage.

The surveys included open-ended items to elicit participants’ likes and dislikes regarding the DA (Supplementary Table S8, see online supplementary material for a color version of this table). When asked what they liked about the DA, there were 57 responses. Participants noted that it was easy to use (*n* = 22/57, 38.5%) and contained clear and helpful information (*n* = 17/57, 29.8%). Some participants also commented that having both text and videos was helpful (*n* = 6/57, 10.5%) and that using the electronic DA was convenient (*n* = 5/57, 8.8%). Participants noted implementation and content improvements, including removing some content from dropdowns, using more graphics, and completing the decision aid alongside a genetic counselor.

### Provider experience

There were 26 oncology providers (16 physicians and 10 nurse practitioners) who consented participants for genetic testing after they completed the DA. Nurse practitioners saw an average of 4.0 participants each, and physicians saw an average of 3.25 participants each (Supplementary Table S9, see online supplementary material for a color version of this table). When asked to complete a provider survey after a participant completed the DA, 13/26 consenting providers completed the survey at least once for up to 45 unique responses (Table 4). In 40/44 (91%) responses, providers indicated their patients were “extremely” or “very” well informed about their testing options and possible outcomes. Most responses (40/45, 89%) indicated that the length of the visit was “about normal,” with two reports of “shorter than normal” visits and only three reports of “longer than normal” visits. All responses indicated that the provider was either “extremely satisfied” (*n* = 18; 41%) or “very satisfied” (*n* = 26; 59%) with the visit. Institution-specific data are shown in Supplementary Table S10 (see online supplementary material for a color version of this table).

**Table 4:** Responses from oncology providers after patients completed the decision aid.

	Total responses	Responses from ovarian cancer providers	Responses from pancreatic cancer providers	*P*-value
	Count (%)	Count (%)	Count (%)	
**1. How informed did you feel the patient was about the testing options and possible outcomes?**	*n* = 44	*n* = 32	*n* = 12	.065
Extremely well informed	14 (31.8%)	10 (31.3%)	4 (33.3%)	
Very well informed	26 (59.1%)	21 (65.6%)	5 (41.7%)	
Somewhat informed	4 (9.1%)	1 (3.1%)	3 (25.0%)	
Not at all informed	0 (0%)	0 (0%)	0 (0%)	
**2. Which multi-gene panel option did the patient prefer?**	*n* = 44	*n* = 32	*n* = 12	.328
Option 1	6 (13.6%)	5 (15.6%)	1 (8.3%)	
Option 2	13 (29.5%)	11 (34.4%)	2 (16.7%)	
Option 3	25 (56.8%)	16 (50.0%)	9 (75.0%)	
**3. How would you rate the length of the visit?**	*n* = 45	*n* = 32	*n* = 13	.319
Longer than normal	3 (6.7%)	3 (9.4%)	0 (0%)	
About normal	40 (88.9%)	27 (84.4%)	13 (100%)	
Shorter than normal	2 (4.4%)	2 (6.2%)	0 (0%)	
**4. How satisfied were you with this visit?**	*n* = 44	*n* = 31	*n* = 13	.647
Extremely satisfied	18 (40.9%)	12 (38.7%)	6 (46.2%)	
Very satisfied	26 (59.1%)	19 (61.3%)	7 (53.8%)	
Somewhat satisfied	0 (0%)	0 (0%)	0 (0%)	
Not at all satisfied	0 (0%)	0 (0%)	0 (0%)	
**5. I received the final printout of the patient’s preferences for genetic testing.**	*n* = 45	*n* = 32	*n* = 13	.023
Yes	43 (95.6%)	32 (100%)	11 (84.6%)	
No	2 (4.4%)	0 (0%)	2 (15.4%)	
**6. I understood the final printout of the patient’s preferences for genetic testing.**	*n* = 45	*n* = 32	*n* = 13	.023
Yes	43 (95.6%)	32 (100%)	11 (84.6%)	
No	2 (4.4%)	0 (0%)	2 (15.4%)	
**7. Obtaining consent and ordering the genetic testing was straightforward**	*n* = 45	*n* = 32	*n* = 13	–
Yes	45 (100%)	32 (100%)	13 (100%)	
No	0 (0%)	0 (0%)	0 (0%)	
**8. I was able to answer questions that my patient had about genetic testing**	*n* = 42	*n* = 29	*n* = 13	.332
Yes	40 (95.2%)	27 (93.1%)	13 (100%)	
No	2 (4.8%)	2 (6.9%)	0 (0%)	
**9. The time it took to use the decision aid interfered with clinic flow**	*n* = 45	*n* = 32	*n* = 13	.742
Yes	9 (20.0%)	6 (18.8%)	3 (23.1%)	
No	36 (80.0%)	26 (81.2%)	10 (76.9%)	

When asked specifically about obtaining consent and ordering the genetic test themselves, all providers responded that the process was straightforward (Table 4). There were two instances where a provider indicated they could not answer the participant’s questions about genetic testing, and both cases were in the ovarian cancer group. Of the 45 survey responses, there were 9 (20%) instances where providers suggested that using the DA interfered with clinic flow. In the clinic, the main disruptions noted by study staff were the timing of the blood draw for genetic testing and minor delays due to the patient still completing the DA when the provider was ready to begin the visit.

In an open-ended question about what providers liked about the DA, there were 18 responses. Several indicated that the process was easy and created minimal additional burden for providers (*n* = 3/18, 17%) and that it was an efficient use of the patient’s time (*n* = 5/18, 28%) (Supplementary Table S11, see online supplementary material for a color version of this table). Providers also noted that the DA supported patient understanding (*n* = 5/18, 28%) and helped patients access services (*n* = 3/18, 17%). Feedback from providers who completed the provider survey included recommendations for ways the patient could complete the DA ahead of their clinic visit, and offering written instructions for patients less familiar with using an iPad.

## Discussion

This study sought to assess the feasibility and acceptability of implementing an electronic DA to support ovarian and pancreatic cancer patients’ genetic testing decision-making in the pre-test setting. Overall, we found it feasible for patients to use the DA in the clinic to support their decision-making process. Participants were able to complete the DA in the time they spent waiting in the clinic for their oncology appointment, and using the DA did not increase the providers’ perceptions of the length of their appointment. Only 5.4% of the participants reported technical difficulties using the DA. Further, after using the DA, most patients (96.7%) were able to make a testing decision with their oncology provider. Only three participants were referred to or requested a session with a genetic counselor to discuss testing in more detail.

We also found that both patients and providers felt that using the DA was acceptable. Participants found the DA helped them make their decision (92.6%), and most noted that they would recommend the DA to a friend or family member to decide about genetic testing (95.1%). Providers also indicated that participants were well-informed about their options (89%). The providers’ satisfaction with the process further supports the DA’s acceptability in this setting.

There was an overall increase in knowledge scores, but there were certain topics where knowledge scores were below 50%. As a pilot study, the goal was to use this information to determine which topics may not be covered clearly and then revise and refine them for use in a future randomized trial between the DA and traditional genetic counseling. Some of the knowledge questions were straightforward, and others were intentionally more challenging, and this enabled us to understand the breadth and depth of knowledge gained. The impact of knowledge gained will be formally assessed when an updated version of the DA is compared to traditional genetic counseling in a future randomized trial.

Overall, nearly all participants proceeded with genetic testing (94.6%). The goal of the DA was to provide balanced and accurate information on the risks and benefits of testing and allow the patient to decide. One-third felt that the DA encouraged genetic testing, suggesting there may be some implied bias toward genetic testing. However, the majority (two-thirds) reported that the information was presented in a balanced manner. It is important to recognize that there are many other factors (e.g. provider recommendations, consensus guidelines, patient support groups) that also contribute to the decision to pursue genetic testing, and most notable are national practice guidelines that currently recommend genetic testing for all patients with pancreatic and ovarian cancer. In this context, the DA may have helped to facilitate uptake, but for other cancer types where universal genetic testing is not recommended, the DA could play a larger role to support decision-making.

To address the growing demand for genetic testing, different service delivery models (SDMs) are being explored, including those that focus on non-genetics providers obtaining pre-test informed consent. Prior studies have shown that leading barriers for non-genetics providers to offer genetic testing are their limited genetics knowledge, low confidence in engaging in discussions about genetics, lack of time for genetics discussions, and uncertain perception of the risks versus benefits of testing [19].

The DA strengthened the ability of non-genetics providers to counsel patients in the pre-test setting by minimizing the burden on providers without compromising patients’ informed decision-making. Once a staff member introduces the DA to the patient, the tool is self-paced, flexible, and can be completed without a provider present. In contrast to educational materials that passively provide information about genetic testing, this decision aid is interactive. It also assists patients with two crucial decisions: whether to pursue genetic testing and which multi-gene panel to select. Most alternative SDMs to date offered a single multi-gene panel option, while our DA offered three distinct choices to patients. Our data indicate there is a wide range of preferences, underscoring the need for a more personalized approach to genetic testing rather than a “one-size-fits-all” model. Furthermore, our findings suggest that the implementation of this DA in the clinic for routine use could be accomplished. Only 5% of participants reported any technical difficulties with the DA, and the training required for established clinic personnel to administer the DA would be minimal. Back-up IT support would be another component for successful implementation.

This study had some limitations. Notably, the overwhelming majority of patients were enrolled at one site (MGH). The small sample size at the BMC site precludes meaningful statistical comparisons between sites. However, important differences in the rate of uptake of the DA when offered were noted (82% at MGH and 25% at BMC). BMC serves a more ethnically, culturally, and linguistically diverse population, and a better understanding of the barriers to DA uptake in different patient populations and hospital settings is needed. There may also be differences in comfort and familiarity with electronic tools. In addition, attitudes, perceptions, and existing knowledge about genetic testing and inherited cancer syndromes can vary in the population, so evaluating the tool in larger, diverse populations is essential [20]. Many individuals who completed the decision aid did not complete all study surveys. The study enrolled participants exclusively at academic medical institutions with site-specific oncology care and providers who specialize in seeing patients with ovarian or pancreatic cancer. These providers may have more experience and knowledge that would facilitate the integration of the DA into the clinic. Finally, the performance of the electronic DA was not compared to the current standard of in-person genetic counseling, so its effectiveness as a tool for pre-test counseling was not formally ascertained in this study.

In conclusion, an electronic decision aid is a feasible and acceptable tool for supporting providers in pre-test education and genetic testing consent for patients with ovarian and pancreatic cancer, but there can be differences in uptake of the tool in disparate populations and clinical settings that may impact broad implementation. Future studies with this DA will seek to formally define its performance and effectiveness when compared directly to traditional genetic counseling.

## Supplementary Material

vmag026_Supplementary_Data

## Data Availability

Data are available by request to corresponding author.

## Appendix 1

**Decision Aid Knowledge Survey** 

Ovarian Cancer

1. If 100 people with ovarian cancer have genetic testing, about how many will have a gene mutation found?

a. 1

b. 20

c. 50

d. 90

2. If a genetic test finds a gene mutation, what might that mean?

a. The cancer has spread

b. The cancer cannot be treated by chemotherapy

c. There may be higher risks of cancers other than ­ovarian cancer

d. There may be a higher risk of dementia

3. Who is potentially impacted by the findings of a genetic test?

a. Patient only

b. Patient and immediate family only

c. Patient and all blood relatives (children, siblings, and cousins)

4. A genetic test may find a change in a gene that is reported as an uncertain result. What is an uncertain result?

a. A result that explains the cause of my ovarian cancer diagnosis

b. A result that tells me about my future risk for cancer

c. A result that will change my treatment plan for ­ovarian cancer

d. A result that does not have any clear impact on me at this time

5. How often does genetic testing lead to an uncertain result?

a. Never

b. Sometimes

c. Often

d. Always

6. Which of the following is an example of a surprise or ­incidental finding?

a. Test finds a mutation that suggests a higher risk of thyroid cancer

b. Test finds a mutation that suggests a genetic cause for ovarian cancer

c. Test finds no mutations

The following questions (questions 7-10) refer to these three genetic test options:

Ovary gene test: a small panel of genes linked to ovarian cancer

Common cancer gene test: a medium panel of genes linked to ovarian cancer and common cancers

Broad cancer gene test: a large panel of genes linked to ­ovarian cancer, common cancers, and rare cancers. This panel also includes newer cancer genes.

 7. Which test will give the most information to guide your ovarian cancer treatment?

a. Ovary gene test

b. Common cancer gene test

c. Broad cancer gene test

d. All three tests provide the same information for ­ovarian cancer treatment

 8. Which test will give the most information about your chance of getting other types of cancer?

a. Ovary gene test

b. Common cancer gene test

c. Broad cancer gene test

d. All three tests provide the same information about other cancers

 9. Which test has the highest chance of finding an ­uncertain result?

a. Ovary gene test

b. Common cancer gene test

c. Broad cancer gene test

d. All three tests have the same chance of ­uncertain results

10. Why might someone select the broad cancer gene test over the ovary gene test?

a. To learn about other genes that may increase the risk of other cancers

b. To learn about additional treatment options for ­ovarian cancer

c. To lower the chance of getting uncertain results

d. To avoid learning things that might impact your family

## Appendix 2

**Decision Aid Knowledge Survey** 

Pancreatic Cancer

1. If 100 people with pancreatic cancer have genetic testing, about how many will have a gene mutation found?

a. 2—4

b. 10—15

c. 50—55

d. 85—90

2. If a genetic test finds a gene mutation, what might that mean?

a. The cancer has spread

b. The cancer cannot be treated by chemotherapy

c. There may be higher risks of cancers other than ­pancreatic cancer

d. There may be a higher risk of dementia

3. Who is potentially impacted by the findings of a genetic test?

a. Patient only

b. Patient and immediate family only

c. Patient and all blood relatives (children, siblings, and cousins)

4. A genetic test may find a change in a gene that is reported as an uncertain result. What is an uncertain result?

a. A result that explains the cause of my pancreatic cancer diagnosis

b. A result that tells me about my future risk for cancer

c. A result that will change my treatment plan for pancreatic cancer

d. A result that does not have any clear impact on me at this time

5. How often does genetic testing lead to an uncertain result?

a. Never

b. Sometimes

c. Often

d. Always

6. Which of the following is an example of a surprise or incidental finding?

a. Test finds a mutation that suggests a higher risk of thyroid cancer

b. Test finds a mutation that suggests a genetic cause for pancreatic cancer

c. Test finds no mutations

**The following questions (questions 7-10) refer to these three genetic test options:** 

Pancreatic cancer gene test: a small panel of genes linked to pancreatic cancer

Common cancer gene test: a medium panel of genes linked to pancreatic cancer and common cancers

Broad cancer gene test: a large panel of genes linked to pancreatic cancer, common cancers, and rare cancers. This panel also includes newer cancer genes.

 7. Which test will give the most information to guide your pancreatic cancer treatment?

a. Pancreatic cancer gene test

b. Common cancer gene test

c. Broad cancer gene test

d. All three tests provide the same information for pancreatic cancer treatment

 8. Which test will give the most information about your chance of getting other types of cancer?

a. Pancreatic cancer gene test

b. Common cancer gene test

c. Broad cancer gene test

d. All three tests provide the same information about other cancers

 9. Which test has the highest chance of finding an uncertain result?

a. Pancreatic cancer gene test

b. Common cancer gene test

c. Broad cancer gene test

d. All three tests have the same chance of uncertain results

10. Why might someone select the broad cancer gene test over the pancreatic cancer gene test?

a. To learn about other genes that may increase the risk of other cancers

b. To learn about additional treatment options for pancreatic cancer

c. To lower the chance of getting uncertain results

d. To avoid learning things that might impact your family

## Appendix 3

**Patient survey.** 

Thank you for using this electronic decision aid for genetic testing. We would appreciate your feedback.

1. Please rate **the way** that the information below was presented in the decision aid (*circle one response*):

- Genetic testing is a blood test that looks for mutations in genes that can increase cancer risk.

   *poor fair good excellent*

- Genetic test results may help guide my treatment options.

   *poor fair good excellent*

- Genetic test results may help me understand the future risks of other cancers.

   *poor fair good excellent*

- Genetic test results may increase stress to me and my family members.

   *poor fair good excellent*

- There are 3 gene panel options that include either a small, medium, or large number of genes:

   *poor fair good excellent*

- Gene test results may come back as positive, negative or uncertain.

   *poor fair good excellent* 

2. The length of the decision aid tool was (*check one):*

Too long  Too short  Just right

3. The amount of information was (*check one*):

Too much information  Too little information Just right

4. I thought that the information presented (*check one*):

Encouraged genetic testing Discouraged genetic testing Was balanced

5. Did you find the decision aid to be helpful in making your decision about genetic testing?

Yes   No

Comments:

6. Was the final summary of your preferences accurate and clear to you?

Yes   No

Comments:

7. Did you have any technical difficulties using the iPad?

Yes  No

Comments:

8. What did you like about the Decision Aid?

9. What suggestions do you have to improve the Decision Aid?

10. Would you recommend this Decision Aid to friends who are interested in genetic testing?

Yes No

Thank you for your participation!!

## Appendix 4

**Provider survey.** 

Thank you for using this electronic decision aid for genetic testing. We would appreciate your feedback.

1. How informed did you feel the patient was about the testing options and possible outcomes?

Extremely well informed

Very well informed

Somewhat informed

Not at all informed 

2. Which option did the patient prefer?

Option 1 (small panel)

Option 2 (medium panel)

Option 3 (large panel)

3. How would you rate the length of the visit?

Shorter than normal

About normal

Longer than normal

4. How satisfied were you with this visit?

Extremely satisfied

Very satisfied

Somewhat satisfied

Not at all satisfied

5. I received the final printout of the patient’s preferences for genetic testing.

Yes   No

6. I understood the final printout about the patient’s preferences for genetic testing.

Yes    No

7. Obtaining consent and ordering the genetic testing was straightforward.

Yes   No

8. I was able to answer questions that my patient had about genetic testing.

Yes   No

9. The time it took to use the Decision Aid interfered with clinic flow.

Yes   No

10. What did you like about the electronic Decision Aid?

11. What suggestions do you have to improve the Decision Aid?

12. Were there any questions from your patient that should have been addressed in the Decision Aid?

Thank you!
