# Supplementary material for: Feasibility and acceptability of an electronic decision aid for genetic testing in ovarian and pancreatic cancer patients
Source: Res Connect. 2026 Apr 28;1(2):vmag026. doi: 10.1093/rescon/vmag026 (PMC13123761; doi:10.1093/rescon/vmag026)
Supplement: vmag026_Supplementary_Data [file vmag026_supplementary_data.docx]

| **Supplementary Table S1**. *Genes on each multi-gene panel offered to ovarian and pancreatic cancer patients* | | | |
| --- | --- | --- | --- |
| **Panels Offered to Ovarian Cancer Patients** | | | |
| **Ovarian Cancer Panel^*^** | | **Common Cancer Panel^†^** | **Broad Cancer Panel^‡^** |
| *14 Genes* | | *36 Genes* | *85 Genes* |
| BRCA1, BRCA2, BRIP1, DICER1, EPCAM, MLH1, MSH2, MSH6, PALB2, PMS2, RAD51C, RAD51D, SMARCA4, STK11 | | All genes included on ovarian cancer panel plus: APC, ATM, AXIN2, BARD1, BMPR1A, CDH1, CDK4, CDKN2A, CHEK2, GREM1, HOXB13, MSH3, MUTYH, NBM, NF1, NTHL1, POLD1, POLE, PTEN, RECQL, SMAD4, TP53 | All genes included on the common cancer panel plus: ADKN1B, AIP, ALK, BAP1, BLM, CDC73, CRCC2, CTNNA1, EGFR, EGLN1, FAM175A (ABRAXAS1), FANCC, FH, FLCN, GALNT12, KIF1B, KIT, LZTR1, MAX, MEN1, MET, MITF, MLH3, MRE11A, NF1, NHL, PALLD, PDGFRA, PHOXB2M, POT1, PRKAR1A, PTCH1, RAD50, RB1, RET, RINT1, RPS20, SDHA, SDHAF1, SDHB, SDHC, SDHD, SMARCB1, SMARCE1, SUFU, TERT, TMEM127, TSC1, TSC2 |
| ** The ovarian cancer panel used was a custom Ambry panel* | | | |
| *† The common cancer panel used was Ambry's CancerNext Panel* | | | |
| *‡ The broad cancer panel used was Ambry's CustomNext-Cancer Panel without pancreatitis genes.* | | | |
| **Panels Offered to Pancreatic Cancer Patients** | | | |
| **Pancreatic Cancer Panel^*^** | **Common Cancer Panel^†^** | | **Broad Cancer Panel^‡^** |
| *20 Genes* | *47 Genes* | | *84 Genes* |
| APC, ATM, BMPR1A, SMAD4, BRCA1, BRCA2, PALB2, CDKN2A, MLH1, MSH2, MSH6, PMS2, EPCAM, STK11, TP53, MEN1, NF1, TSC1, TSC2, VHL | All genes included on pancreatic cancer panel plus: AXIN2, BARD1, BRIP1, CDH1, CDK4, CHEK2, CTNNA1, DICER1, GREM1, HOXB13, KIT, MSH3, MUTYH, NBN, NTHL1, PDGFRA, POLD1, POLE, PTEN, RAD50, RAD51C, RAD51D, SDHA, SDHB, SDHC, SMAD4, SMARCA4 | | All genes included on the common cancer panel plus: AIP, ALK, BAP1, BLM, CASR, CDC73, CDKN1B, CDKN1C, CEBPA, DIS3L2, EGFR, FH, FLCN, GATA2, GPC3, HRAS, MAX, MET, MITF, NF2, PHOX2B, POT1, PRKAR1A, PTCH1, RB1, RECQL4, RET, RUNX1, SDHAF2, SMARCB1, SMARCE1, SUFU, TERC, TMEM127, TERT, WRN, WT1 |
| ** The pancreatic cancer panel used was Invitae’s Hereditary Pancreatic Cancer Panel* | | | |
| *† The common cancer panel used was Invitae's Common Hereditary Cancers Panel* | | | |
| *‡ The broad cancer panel used was Invitae’s Multi-Cancer Panel.* | | | |

| **Supplementary Table S2.** *Demographics of participants at BMC (n=4)* | |
| --- | --- |
| **Age (Years)** |  |
| <50 | 1 (25%) |
| 50-70 | 3 (75%) |
| >70 | - |
|  |  |
| **Gender** |  |
| Male | 2 (50%) |
| Female | 2 (50%) |
|  |  |
| **Race** |  |
| White | - |
| Black or African American | 2 (50%) |
| Asian | - |
| Declined/Other | 2 (50%) |
|  |  |
| **Ethnicity** |  |
| Non-Hispanic or Latino | 2 (50%) |
| Hispanic or Latino | 2 (50%) |
| Other | - |
|  |  |
| **Cancer Stage** |  |
| I | - |
| II | - |
| III | 2 (50%) |
| IV | 2 (50%) |
|  |  |
| **Cancer Type** |  |
| Ovarian Cancer | 1 (25%) |
| Pancreatic Cancer | 3 (75%) |

| **Supplementary Table S3**. *Participant responses to values statements within the Decision Aid by cancer type* | | | | |
| --- | --- | --- | --- | --- |
|  | Mean Score (SD) | | | *p*-Value* |
|  | All Participants | Ovarian Cancer Patients | Pancreatic Cancer Patients |  |
| **1. Getting genetic information that may be useful to me would be:** | **n=83** | **n=40** | **n=43** | 0.525 |
| *(1: Very difficult for me right now < -- > 7: Important to me, even if it caused stress)* | 6.3 (1.0) | 6.2 (1.0) | 6.3 (0.9) |  |
| **2. Getting results that are not helpful is:** | **n=83** | **n=40** | **n=43** | 0.929 |
| *(1: Something I am concerned about < -- > 7: Not a concern)* | 5.0 (2.0) | 5.0 (2.1) | 5.0 (2.0) |  |
| **3. Getting information that may guide my treatment is:** | **n=83** | **n=40** | **n=43** | 0.561 |
| *(1: Not important to me < -- > 7: Very important to me)* | 6.8 (0.6) | 6.8 (0.6) | 6.8 (0.6) |  |
| **4. Understanding my risk for other cancers is:** | **n=82** | **n=39** | **n=43** | 0.011 |
| *(1: Not important to me < -- > 7: Very important to me)* | 6.5 (0.9) | 6.8 (0.5) | 6.3 (1.1) |  |
| **5. Having my genetic testing results part of my medical records is something:** | **n=83** | **n=40** | **n=43** | 0.123 |
| *(1: I am very concerned about < -- > 7: I am not very concerned about)* | 5.4 (1.9) | 5.1 (2.1) | 5.8 (1.7) |  |
| **6. The risk of possible genetic discrimination is something I am:** | **n=82** | **n=39** | **n=43** | 0.084 |
| *(1: Very worried about < -- > 7: Not very worried about)* | 5.6 (1.8) | 5.2 (2.1) | 5.9 (1.3) |  |
| **7. Understanding why I got cancer is:** | **n=82** | **n=39** | **n=43** | 0.226 |
| *(1: Not important to me < -- > 7: Very important to me)* | 6.5 (0.9) | 6.6 (0.7) | 6.4 (1.0) |  |
| **8. Getting information that may be valuable to my family’s health is:** | **n=82** | **n=39** | **n=43** | 0.284 |
| *(1: Not important to me < -- > 7: Very important to me)* | 6.8 (0.5) | 6.9 (0.3) | 6.7 (0.7) |  |
| **9. Getting information that may upset or cause stress to my family members would be:** | **n=82** | **n=39** | **n=43** | 0.200 |
| *(1: Very difficult for them to deal with < -- > 7: Something we could handle)* | 5.8 (1.5) | 6.0 (1.3) | 5.6 (1.6) |  |
| **10. The possible cost of testing (including co-pays, deductibles, etc.) is:** | **n=82** | **n=40** | **n=42** | 0.907 |
| *(1: Something I am very concerned about < -- > 7: Not something I am concerned about)* | 4.6 (2.1) | 4.6 (2.1) | 4.7 (2.2) |  |
| **Total Average Rating** *(n=83)* | **n=83** | **n=40** | **n=43** | 0.689 |
|  | 5.9  (0.9) | 5.8  (1.0) | 5.9  (0.8) |  |
| **Adjusted for cancer stage.* | | | | |

->

| **Supplementary Table S4**. *Participant responses to values statements within the Decision Aid by institution* | | | |
| --- | --- | --- | --- |
|  | Mean Score (SD) | | |
|  | All Participants | MGH | BMC |
| **1. Getting genetic information that may be useful to me would be:** | **n=83** | **n=79** | **n=4** |
| *(1: Very difficult for me right now < -- > 7: Important to me, even if it caused stress)* | 6.3 (1.0) | 6.3 (0.9) | 6.3 (1.5) |
| **2. Getting results that are not helpful is:** | **n=83** | **n=79** | **n=4** |
| *(1: Something I am concerned about < -- > 7: Not a concern)* | 5.0 (2.0) | 5.1 (2.0) | 3.0 (2.8) |
| **3. Getting information that may guide my treatment is:** | **n=83** | **n=79** | **n=4** |
| *(1: Not important to me < -- > 7: Very important to me)* | 6.8 (0.6) | 6.8 (0.6) | 7.0 (0.0) |
| **4. Understanding my risk for other cancers is:** | **n=82** | **n=78** | **n=4** |
| *(1: Not important to me < -- > 7: Very important to me)* | 6.5 (0.9) | 6.4 (1.2) | 7.0 (0.0) |
| **5. Having my genetic testing results part of my medical records is something:** | **n=83** | **n=79** | **n=4** |
| *(1: I am very concerned about < -- > 7: I am not very concerned about)* | 5.4 (1.9) | 5.6 (1.8) | 2.5 (2.4) |
| **6. The risk of possible genetic discrimination is something I am:** | **n=82** | **n=78** | **n=4** |
| *(1: Very worried about < -- > 7: Not very worried about)* | 5.6 (1.8) | 5.4 (1.9) | 7.0 (0.0) |
| **7. Understanding why I got cancer is:** | **n=82** | **n=78** | **n=4** |
| *(1: Not important to me < -- > 7: Very important to me)* | 6.5 (0.9) | 6.4 (1.2) | 7.0 (0.0) |
| **8. Getting information that may be valuable to my family’s health is:** | **n=82** | **n=78** | **n=4** |
| *(1: Not important to me < -- > 7: Very important to me)* | 6.8 (0.5) | 6.7 (0.9) | 7.0 (0.0) |
| **9. Getting information that may upset or cause stress to my family members would be:** | **n=82** | **n=78** | **n=4** |
| *(1: Very difficult for them to deal with < -- > 7: Something we could handle)* | 5.8 (1.5) | 5.7 (1.6) | 5.8 (1.3) |
| **10. The possible cost of testing (including co-pays, deductibles, etc.) is:** | **n=82** | **n=78** | **n=4** |
| *(1: Something I am very concerned about < -- > 7: Not something I am concerned about)* | 4.6 (2.1) | 4.7 (2.1) | 3.0 (2.7) |
| **Total Average Rating** | **n=83** | **n=79** | **n=4** |
|  | 5.9 (0.9) | 5.9 (0.9) | 5.6 (0.9) |

| **Supplementary Table S5.** *Frequency of correct responses to knowledge survey questions completed by participants before and after using the Decision Aid* |
| --- |

|  | Percent Correct | | *p*-value* |
| --- | --- | --- | --- |
|  | Baseline  (n=52) | Follow Up  (n=52) |  |
| Q1. If 100 people with ovarian/pancreatic cancer have genetic  testing, about how many will have a gene mutation found? | 58% | 69% | 0.210 |
| Q2. If a genetic test finds a gene mutation, what might that mean? | 88% | 90% | 1.000 |
| Q3. Who is potentially impacted by the findings of a genetic test? | 85% | 90% | 0.375 |
| Q4. A genetic test may find a change in a gene that is reported as  an uncertain result. What is an uncertain result? | 62% | 83% | <0.001 |
| Q5. How often does genetic testing lead to an uncertain result? | 79% | 92% | 0.065 |
| Q6. Which of the following is an example of a surprise or incidental  finding? | 25% | 40% | 0.057 |
| Q7. Which test will give the most information to guide your  ovarian/pancreatic cancer treatment? | 33% | 40% | 0.541 |
| Q8. Which test will give the most information about your chance of  getting other types of cancer? | 60% | 73% | 0.143 |
| Q9. Which test has the highest chance of finding an uncertain  result? | 40% | 56% | 0.134 |
| Q10. Why might someone select the broad cancer gene test over  the ovary/pancreas gene test? | 75% | 88% | 0.065 |

** McNemar’s tests*

| **Supplementary Table S6:** *Participant ratings of the information presented in the Decision Aid* | | | | | |
| --- | --- | --- | --- | --- | --- |
| **Question** | **Rating** | **Total  Participants** | **Ovarian  Cancer Patients** | **Pancreatic  Cancer Patients** | ***p*-value*** |
|  | | **n=88** | **n=47** | **n=41** |  |
| **Q1a. Genetic testing is a blood test that looks for mutations in genes that can increase cancer risk** | **1.Poor** | 1.1% | . | 2.4% | 0.944 |
|  | **2.Fair** | 3.4% | . | 7.3% |  |
|  | **3.Good** | 33.0% | 25.5% | 41.5% |  |
|  | **4.Excellent** | 62.5% | 74.5% | 48.8% |  |
|  | | **n=87** | **n=46** | **n=41** |  |
| **Q1b. Genetic test results may help guide my treatment options** | **1.Poor** | 2.3% | . | 4.9% | 0.084 |
|  | **2.Fair** | 4.6% | 2.2% | 7.3% |  |
|  | **3.Good** | 34.5% | 23.9% | 46.3% |  |
|  | **4.Excellent** | 58.6% | 73.9% | 41.5% |  |
|  | | **n=88** | **n=47** | **n=41** |  |
| **Q1c. Genetic test results may help me understand the future risks of other cancers** | **1.Poor** | 1.1% | . | 2.4% | 0.364 |
|  | **2.Fair** | 4.5% | 2.1% | 7.3% |  |
|  | **3.Good** | 29.5% | 25.5% | 34.1% |  |
|  | **4.Excellent** | 64.8% | 72.3% | 56.1% |  |
|  | | **n=87** | **n=46** | **n=41** |  |
| **Q1d. Genetic test results may increase stress to me and my family members** | **1.Poor** | 5.7% | 6.5% | 4.9% | 0.253 |
|  | **2.Fair** | 9.2% | 4.3% | 14.6% |  |
|  | **3.Good** | 37.9% | 37.0% | 39.0% |  |
|  | **4.Excellent** | 47.1% | 52.2% | 41.5% |  |
|  | | **n=87** | **n=46** | **n=41** |  |
| **Q1e. There are 3 gene panel options that include either a small, medium, or large number of genes** | **1.Poor** | 2.3% | 2.2% | 2.4% | 0.932 |
|  | **2.Fair** | 4.6% | 4.3% | 4.9% |  |
|  | **3.Good** | 35.6% | 34.8% | 36.6% |  |
|  | **4.Excellent** | 57.5% | 58.7% | 56.1% |  |
|  | | **n=86** | **n=45** | **n=41** |  |
| **Q1f. Gene test results may come back as positive, negative or uncertain** | **1.Poor** | 1.2% | . | 2.4% | 0.938 |
|  | **2.Fair** | 7.0% | 6.7% | 7.3% |  |
|  | **3.Good** | 38.4% | 35.6% | 41.5% |  |
|  | **4.Excellent** | 53.5% | 57.8% | 48.8% |  |

* Rating dichotomized as (poor/fair vs. good/excellent), adjusting for cancer stage.

| **Supplementary Table S7:** *Participant responses to the patient survey about their experience using the Decision Aid by institution* | | | | |
| --- | --- | --- | --- | --- |
| **Question** | **Rating** | **Total  Participants** | **MGH** | **BMC** |
|  | | **n=86** | **N=83** | **N=3** |
| **The length of the decision aid tool was:** | **Too Short** | 2 (2.3%) | 2 (2.4%) | - |
|  | **Just right** | 82 (95.3%) | 79 (95.2%) | 3 (100%) |
|  | **Too Long** | 2 (2.3%) | 2 (2.4%) | - |
|  | | **n=84** | **N=81** | **N=3** |
| **The amount of information was:** | **Too Little information** | 4 (4.8%) | 4 (4.9%) | - |
|  | **Just Right** | 77 (91.7%) | 74 (91.4%) | 3 (100%) |
|  | **Too Much Information** | 3 (3.6%) | 3 (3.7%) | - |
|  | | **n=83** | **N=80** | **N=3** |
| **I thought the information presented:** | **Discouraged Genetic Testing** | - | - | - |
|  | **Was Balanced** | 55 (66.3%) | 54 (67.5%) | 1 (33.3%) |
|  | **Encouraged Genetic Testing** | 28 (33.7%) | 26 (32.5) | 2 (66.7%) |
|  | | **n=81** | **N=78** | **N=3** |
| **Did you find the Decision Aid to be helpful in making your decision about genetic testing?** | **Yes** | 75 (92.6%) | 72 (92.3%) | 3 (100%) |
|  | **No** | 6 (7.4%) | 6 (7.7%) | - |
|  | | **n=81** | **N=78** | **N=3** |
| **Was the final summary of your preferences accurate and clear to you?** | **Yes** | 77 (95.1%) | 74 (94.9%) | 3 (100%) |
|  | **No** | 4 (4.9%) | 4 (5.4%) | - |
|  | | **n=81** | **N=78** | **N=3** |
| **Would you recommend this Decision Aid to friends who are interested in genetic testing?** | **Yes** | 77 (95.1%) | 74 (94.9%) | 3 (100%) |
|  | **No** | 4 (4.9%) | 4 (5.1%) | - |

| **Supplementary Table S8**. *Qualitative feedback from participants after using the Decision Aid* | | |
| --- | --- | --- |
|  | **Theme (# of Responses)** | **Examples of Responses** |
| **Did you find the decision aid to be helpful in making your decision about genetic testing? (comments)** | | |
|  | Helped Confirm I was making the right decision (6) | I had already decided to have genetic testing but the decision aid confirmed this was the right decision for me. |
|  |  | reinforced decision |
|  |  | It confirmed my previous decision but I didn't know there would be options! |
|  |  | I was going to have genetic testing anyway for my kids, but the information was very helpful in choosing + informing me. |
|  |  | I have a daughter whom I don't want to go through what I am. |
|  |  | I had already decided I would be tested. |
|  | Miscellaneous (5) | If I used it yes |
|  |  | She was very nice and informative. |
|  |  | more visual graphics may be helpful |
|  |  | had done research on genetic testing on my own beforehand |
|  |  | However, I did my research ahead of time |
| **What did you like about the Decision Aid?** | | |
|  | Information is Clear and Effective (17) | very clear |
|  |  | All of it was very well done. Videos were clear and concise. |
|  |  | Very clear and very helpful |
|  |  | Very informative (x3) |
|  |  | clean and effectively explained genetic testing options |
|  |  | Enabled informed decision making |
|  |  | very knowledgeable info |
|  |  | Very informative and concise |
|  |  | Offers info to help |
|  |  | It was efficient and to the point / didn't take long |
|  |  | It was clear and direct - multiple ways to get info. |
|  |  | liked how it explained not too much medical language spoke right to pt |
|  |  | Contained information that was helpful |
|  |  | The information was clean concise and complete. |
|  |  | gave me the information necessary to make the decision |
|  | The DA is easy to use (22) | easy to follow |
|  |  | short, easy to use & understand |
|  |  | ease of use, concise, legible |
|  |  | simple (x3) |
|  |  | It was easy and assistant counselor (KPW) was excellent |
|  |  | Easy to read (x2) |
|  |  | Clear and easy to understand |
|  |  | Very clear and informative, easy to understand |
|  |  | easy to work through |
|  |  | It was concise & easy to understand |
|  |  | Ease, but prefer paper |
|  |  | ease, info |
|  |  | Simple. Clear. |
|  |  | Ease of Participating |
|  |  | It was easy to navigate and was nice that we could fast forward information we did not find relevant to us. |
|  |  | I thought it was easily understood and informative. |
|  |  | Easy to understand (x3) |
|  | Use of videos and text provides helpful flexibility (6) | It explained everything clearly and the option for video was a great idea. |
|  |  | Being able to read the information as well as watch the video |
|  |  | I liked the option to just read the text rather than have to watch the videos. But it's a great design to allow people to do either or both. |
|  |  | The option to watch a video or read the text |
|  |  | the videos were more helpful |
|  |  | Clarity was spot-on; design and color choice was great/layout etc; beginning video was great, precise |
|  | The DA was convenient to use (5) | Mobile |
|  |  | Clear / At your own pace |
|  |  | Concise and to the point. Right length and amount of info |
|  |  | short |
|  |  | quick |
|  | Miscellaneous (7) | Me gusto toda la informacion y asi saber acerca del examen genetico y tomar mis precauciones en un futuro muy buena informacion. |
|  |  | She was very nice, polite, and informative |
|  |  | I think it would be helpful for others |
|  |  | Neutral (x2) |
|  |  | A piece of mine |
|  |  | nothing to dislike (x2) |
|  |  | Excellent |
| **What suggestions do you have to improve the Decision Aid?** | | |
|  | Implementation Improvements (6) | do survey during infusion |
|  |  | completing it with counselor |
|  |  | I was going to say there should be a pre-test if there's a post-test, but it's true that it may tip people off to pay closer attention to details than they otherwise would. |
|  |  | I was less clear before today, despite discussion with the doctor. Perhaps one email or text that provides an overview ahead of time. That said, we've received a lot of info recently so perhaps I missed it. |
|  |  | need WIFI printed materials as a supplement different languages |
|  |  | Patient wrote: I am 80 I am very low tech I would prefer a real person to a machine |
|  | Content Improvements (11) | Maybe to give more information |
|  |  | Include what's in decision aid in video |
|  |  | Some background on genes/how list derived |
|  |  | Explain scrolling down to next at bottom of page - maybe include this info or an arrow on each page |
|  |  | Clearer options on paper |
|  |  | More time to go through it. Information should be upfront, not in dropdowns |
|  |  | more graphics to support statements |
|  |  | more videos |
|  |  | be more direct |
|  |  | less sales. more hard facts. less Lyndsey |
|  |  | More videos, less difficult to absorb when hearing it |
|  | Miscellaneous (5) | I thought it was a great tool. I hate surveys and this was great and easy to understand. |
|  |  | it's good |
|  |  | It is helpful |
|  |  | For me it's complicated |
|  |  | Decision Aid was very helpful but still had difficulty with the survey |

| **Supplementary Table S9:** *Number of participants seen by each provider by cancer type* | | | | |
| --- | --- | --- | --- | --- |
|  | **Number of Providers** | **Total Number of participants seen by an MD or NP** | **Average number of patients seen per MD or NP** | **Average number of patients seen per provider (total)** |
| **All Participants (n=92)** | | | | |
| **MD** | 16 | 52 | 3.25 | 3.5 |
| **NP** | 10 | 40 | 4 |  |
| **Ovarian Cancer Patients (n=47)** | | | | |
| **MD** | 8 | 22 | 2.75 | 3.9 |
| **NP** | 4 | 25 | 6.25 |  |
| **Pancreatic Cancer Patients (n=45)** | | | | |
| **MD** | 8 | 30 | 3.75 | 3.2 |
| **NP** | 6 | 15 | 2.5 |  |

*No significant difference between Ovarian or Pancreatic Cancer patients (p=0.61)*

| **Supplementary Table S10:** *Number of participants seen by each provider by institution* | | | | |
| --- | --- | --- | --- | --- |
|  | **Number of Providers** | **Total Number of participants seen by an MD or NP** | **Average number of patients seen per MD or NP** | **Average number of patients seen per provider (total)** |
| **All Participants (n=92)** | | | | |
| **MD** | 16 | 52 | 3.25 | 3.5 |
| **NP** | 10 | 40 | 4 |  |
| **MGH (n=88)** | | | | |
| **MD** | 13 | 48 | 3.7 | 3.82 |
| **NP** | 10 | 40 | 4 |  |
| **BMC (n=4)** | | | | |
| **MD** | 3 | 4 | 1.33 | 1.33 |
| **NP** | - | - | - |  |

| **Supplementary Table S11**. *Qualitative feedback from oncology providers after use of the Decision Aid* | |
| --- | --- |
|  |  |
| **Question (# of Responses)** | **Response** |
| What did you like about the electronic Decision Aid? (18) | Patient liked it. Said it was very clear and easy to use. |
|  | Easier for patient and provider |
|  | User friendly provided patient is comfortable using a computer. |
|  | Requires little input from me, making visit efficient |
|  | Pt completed prior to my time with her and pt had no questions or concerns about process |
|  | Quickly gives information to patient |
|  | Reduces time spent on counseling, increases pt satisfaction |
|  | Seamless |
|  | Inform pts |
|  | Informative, patient understands panels |
|  | Pt was well-informed and comfortable with her decision to pursue genetic testing. |
|  | It gauges patient's interest in performing genetic testing. |
|  | Pt well informed. did not have questions |
|  | All information is there for patient without my intervention or interruption |
|  | Eventually we can do in an even more streamlined manner |
|  | Quick access for patients |
|  | Too early to say |
|  | still don't understand it??? |
| What suggestions do you have to improve the Decision Aid? (11) | Too early to say |
|  | None at this time (x2) |
|  | I wonder if there is a way to do this at home prior to visit |
|  | Some documentation in EPIC that DA was completed and that labs were drawn and sent. |
|  | Instructions for iPad-naive users |
|  | If somehow they could complete the DA prior to their visit - or be asked to come in earlier? The time it took for her to complete delayed the nurse, who then had to see another patient, thus was then late to see this patient. Luckily this ran into lunch hour so we didn't run too far behind :) Maybe they could do the DA down in infusion and have the sample drawn at the next visit? |
|  | maybe having it "scheduled" in Epic appts in the future would reduce time genetics needs to send out emails to providers?? |
|  | Too early to say |
|  | None |
|  | Testing kit was not dropped off in advance. |
| Were there any questions from your patient that should have been addressed in the Decision Aid? (15) | She had no additional questions |
|  | Not sure |
|  | Not sure - I may want to take a look at the actual DA at some point |
|  | No, patient had no questions |
|  | No (x8) |
|  | Patient had no questions |
|  | N/A (x2) |
